# miR-301a-mediated crosstalk between the Hedgehog and HIPPO/YAP signaling pathways promotes pancreatic cancer

**DOI:** 10.1080/15384047.2025.2457761

**Published:** 2025-01-23

**Authors:** Bing Qi, Yuqiong Wang, Xian Zhu, Yanfang Gong, Jing Jin, Hongyu Wu, Xiaohua Man, Feng Liu, Wenzhu Yao, Jun Gao

**Affiliations:** a Institute of Oncology, Second Affiliated Hospital, Xi’an Medical University, Xi’an, China; b Department of Gastroenterology, The Hospital of 92608 People’s Liberation Army of China (PLA) Troops, Shanghai, China; c Department of Gastroenterology, Ningbo Medical Center Lihuili Hospital, Ningbo, China; d Department of Gastroenterology, Changhai Hospital, Second Military Medical University, Shanghai, China; e Department of Gastroenterology, Tongchuan People’s Hospital, Tongchuan, China

**Keywords:** miR-301a, Hedgehog, Gli1, Hippo/YAP, MST1(STK4)

## Abstract

Pancreatic ductal adenocarcinoma (PDAC) poses a significant challenge in oncology due to its dismal prognosis and limited therapeutic options. In this study, we investigated the role of miR-301a in facilitating crosstalk between the Hedgehog (Hh) and HIPPO/YAP signaling pathways during the progression of PDAC. Our findings revealed that miR-301a served as a central regulatory node, targeting Gli1 within the Hh pathway and STK4 within the HIPPO/YAP pathway. Immunohistochemical and molecular analyses confirmed dysregulation of pathway components in pancreatic cancer, underscoring the pivotal role of miR-301a. Functional assays demonstrated the impact of miR-301a on cell proliferation and apoptosis, particularly in synergy with TNF-α. Overall, our study elucidated the intricate interplay between the Hh and HIPPO/YAP pathways mediated by miR-301a, providing valuable insights into potential therapeutic strategies for intervening in PDAC.

## GRAPHICAL ABSTRACT

**Figure:**
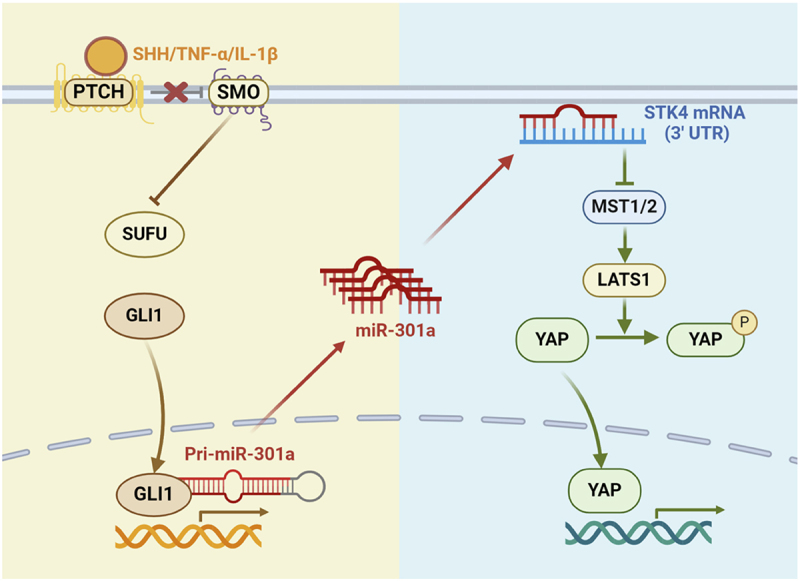

## 1. Introduction

Pancreatic ductal adenocarcinoma (PDAC) presents a formidable challenge in oncology, boasting a dismal 5-year overall survival (OS) rate of less than 10%^1^ and poised to become the second leading cause of cancer-related death by 2030.^2^ Although surgical resection offers a chance for cure, it remains feasible for only a small fraction of patients, leaving the majority to rely on systemic chemotherapy, which often proves ineffective due to PDAC’s notorious resistance.^3^ These grim realities underscore the urgent need for innovative and more efficacious therapies for PDAC patients.^4^

The pathogenesis of PDAC involves a complex interplay of various signaling pathways that regulate key cellular processes such as proliferation, survival, and metastasis.^5^ One of the pivotal pathways implicated in PDAC is the hedgehog (Hh) signaling pathway.^6^ Aberrant activation of the Hh pathway has been observed in PDAC, with both canonical and non-canonical signaling pathways contributing to tumor progression.^6^ Sonic hedgehog (Shh), a key ligand in the Hh pathway, is frequently overexpressed in PDAC, driving tumorigenesis and promoting metastasis. The downstream effector of the Hh pathway, glioma-associated oncogene homolog 1 (Gli1), plays a crucial role in mediating the oncogenic effects of Shh, further emphasizing the significance of Hh signaling in PDAC development.^7^ Notably, reduced levels of Shh and Gli1 proteins correlate with improved survival outcomes in PDAC patients post-resection.^8^

Another important pathway in PDAC is the Hippo-YAP pathway. Dysregulation of this pathway, characterized by aberrant activation of the Yes-associated protein (YAP), has been implicated in PDAC pathogenesis.^9^ Active YAP promotes cancer cell proliferation, survival, and metastasis, contributing to the aggressiveness of PDAC tumors. The core components of the Hippo pathway, including mammalian Ste20-like kinases (MST1/2) and large tumor suppressor kinases (LATS1/2), regulate YAP activity through phosphorylation-mediated mechanisms. In PDAC, the dysregulation of these core components leads to the nuclear accumulation of YAP, where it interacts with transcription factors to promote the expression of genes involved in tumorigenesis.

Moreover, miRNAs, as endogenous post-transcriptional regulators, play crucial roles in modulating biological pathways, including those involved in cancer development and progression.^10^ Their potential as therapeutic targets is underscored by studies demonstrating their modulation by pharmacological agents and their involvement in mediating crosstalk among pathways critical in oncogenesis.^11^ miRNAs have garnered increasing interest as therapeutic tools to target cancer pathways, including those involved in PDAC.^12–14^ Their sequence-specific degradation of target messenger RNAs offers promise, albeit hindered by challenges in clinical translation, such as delivery and stability issues. Downregulation of miR-345 in pancreatic cancer tissues and cell lines has been documented, with its reintroduction showing inhibitory effects on PDAC cell growth in vitro.^15^ Innovative approaches, such as nanoscale delivery systems facilitating co-delivery of miRNAs and chemotherapeutic agents, show promise in overcoming these hurdles.^16^

Emerging evidence highlights the complex interplay between the Hedgehog (Hh) and HIPPO/YAP signaling pathways, both of which play critical roles in tumor progression. The Hh pathway, through effectors such as GLI1, drives cellular proliferation and metastasis, while the HIPPO pathway acts as a tumor suppressor by regulating YAP/TAZ phosphorylation to inhibit their nuclear localization and oncogenic activity. Recent studies^9,17^ have demonstrated that these pathways are interconnected, interacting through shared molecular mediators like GLI1 and YAP/TAZ to form a regulatory loop that reinforces oncogenic signaling. While miRNAs are known to independently regulate either the Hh or HIPPO pathway, our findings show for the first time that miR-301a can simultaneously modulate both. This underscores the need to explore miRNA-mediated mechanisms linking these pathways, revealing novel regulatory networks and therapeutic targets in cancer progression.

In our previous studies, we found cytokines TNF-α and IL-1β can activate the Hh pathway, while also suggesting the involvement of miRNAs in the crosstalk between the Hh and Hippo pathways.^18^ To further elucidate the interplay between key signaling pathways in PDAC, particularly the Hedgehog and HIPPO/YAP pathways, we conducted a comprehensive investigation. We evaluated differentially expressed miRNAs and core proteins of the Hippo pathway in PDAC and adjacent tissues, exploring the potential influence of Hedgehog signaling on the Hippo/YAP pathway via miRNA-mediated mechanisms. Through experimental manipulation of key molecular components and functional assays, for the first time, we have identified miR-301a as a central regulatory node in the Hedgehog and Hippo/YAP signaling pathways. Our findings provide novel insights into the regulatory mechanisms underlying crosstalk between the Hh and HIPPO/YAP pathways, highlighting the intricate network of interactions involved in pancreatic cancer pathogenesis.

## 2. Materials and methods

### 2.1. Cell culture

PDAC cells, including SW1990 and Panc-1, were procured from the Type Culture Collection of the Chinese Academy of Sciences (Shanghai, China). These cells were cultured in Dulbecco’s Modified Eagle Medium (DMEM, Gibco, USA) supplemented with 10% fetal bovine serum (FBS, Gibco, USA) and penicillin-streptomycin solution (100 U/mL penicillin/100 ng/mL streptomycin, Hyclone, USA) at 37°C with 5% CO2. Recombinant human SHH, TNF-α and IL-1β were acquired from R&D Systems, USA. The cells were seeded in T25 culture dishes (Corning, USA). Upon reaching 50% confluency, they were subjected to various treatments, including Shh/TNF-α/IL-1β or Verteporfin (Sigma, USA) for 48 hours, with the medium refreshed every 12 hours. The cells in the logarithmic phase were transfected with miR-301 mimic, miR-301 inhibitor, Gli1 cDNA and their corresponding controls.

### 2.2. RNA extraction and quantitative PCR

To analyze mRNA expression, total RNA was extracted from pancreatic ductal adenocarcinoma (PDAC) cells using Trizol reagent followed by DNase I digestion for 15 minutes to remove genomic DNA contamination. RNA purification was then performed using the RNeasy Kit (Qiagen, Germany). Subsequently, 1 µg of purified RNA was subjected to reverse transcription in a 20 µL reaction system at 37°C for 15 minutes following the manufacturer’s protocol. TaqMan primers targeting Gli1, NF-kB, Shh, KRAS, and GAPDH were obtained from Invitrogen (Shanghai Life Technologies Biotechnology Co., Ltd.). Quantitative PCR (qPCR) was conducted using a 20 µL reaction mixture containing gene-specific TaqMan primers on the Roche LightCycler® 480 instrument (Roche, Switzerland). The expression levels of each target gene were normalized to GAPDH levels in each sample. All experiments were performed in triplicate to ensure accuracy and reproducibility.

### 2.3. Microarray analysis for mRNA and miRNA

To analyze differentially expressed miRNAs and mRNAs in SW1990 and PANC-1 cells, we compared GLI1 overexpression groups with corresponding control groups, each consisting of three samples. Total RNA was extracted using the mirVana™ RNA Isolation Kit (Applied Biosystems p/n AM1556) and quantified with a NanoDrop ND-2000 spectrophotometer. RNA integrity was assessed using an Agilent Bioanalyzer 2100. Subsequently, the extracted RNA was reverse-transcribed into cDNA, synthesized into complementary RNA (cRNA), and labeled with Cyanine-3-CTP. The labeled cRNA was hybridized onto Agilent microarray slides, washed, and scanned using the Agilent Scanner G2505C to capture fluorescence signals. The resulting data underwent background correction, normalization, and differential expression analysis to compare the expression profiles between the Gli1 overexpression and control groups.

### 2.4. Construction of recombinant lentivirus and cell infection

To downregulate the expression of the Gli1 gene, short hairpin RNA (shRNA) constructs were employed, with a non-silencing fragment serving as the negative control. The synthesis of shRNA was outsourced to Shanghai GenePharma Co., Ltd (Shanghai, China). The shRNA fragments were produced through hybridization of synthesized sense and antisense oligonucleotides, and subsequently cloned into the pGLV-U6-EGFP plasmid, yielding the pGLV-Sh-Gli1 plasmid. The integrity of the shRNA cassettes was validated via direct sequencing.

The shRNA-containing plasmid, pGLV-sh-Gli1, in conjunction with essential components for virus packaging (VSVG and Gag/pol/rev plasmid), were co-transfected into SW1990 and PANC-1 cells using Lipofectamine 2000. Following filtration of the harvested medium through 0.45 μm-filters, virus concentration was achieved via centrifugation at 4,000 × g (Eppendorf, Hamburg, Germany) for 15 minutes, succeeded by an additional 2 minutes at 1,000 × g. The concentrated virus was then stored at − 80°C, with lentiviral vector titers determined through dilution assays employing fluorescence microscopy (IX71; Olympus, Tokyo, Japan).

### 2.5. Nuclear and cytoplasmic protein extraction

The cells were harvested using 0.25% trypsin (Gibco, USA) and subsequently centrifuged at 12,000 rpm for 5 minutes. Following three washes with phosphate-buffered saline (PBS), nuclear and cytoplasmic proteins were separately extracted utilizing NE-PER nuclear and cytoplasmic extraction reagents (Thermo, USA), in accordance with the manufacturer’s instructions. The concentration of proteins was determined using the bicinchoninic acid (BCA) protein assay kit (Thermo, USA) as per the provided protocol, with the protein solution subsequently diluted to a concentration of 1 μg/μL.

### 2.6. Western blot

Both nuclear and cytoplasmic proteins were resolved using sodium dodecyl sulfate-polyacrylamide gel electrophoresis (SDS-PAGE) and subsequently transferred onto polyvinylidene fluoride (PVDF) membranes (Bio-Rad, USA). Immunoblotting was conducted overnight at 4°C using the following primary antibodies: rabbit anti-SHH (1:3000, Abcam, USA), rabbit anti-HHIP (1:3000, Abcam, USA), rabbit anti-GLI1 (1:1000, Abcam, USA), rabbit anti-phospho-YAP (Ser127) (1:1000, Abcam, USA), rabbit anti-phospho-YAP (Ser397) (1:1000, Abcam, USA), rabbit anti-YAP (1:200, Santa Cruz, USA), rabbit anti-MST1 (1:1000, Abcam, USA), rabbit anti-MST2 (1:1000, Abcam, USA), rabbit anti-LAST1 (1:1000, Abcam, USA), rabbit anti-β-actin (1:1000, Abcam, USA), and mouse anti-GAPDH (1:2000, Abcam, USA). β-actin and GAPDH served as controls for cytoplasmic and nuclear proteins, respectively. Following incubation with primary antibodies, the blots were visualized using enhanced chemiluminescence reagent (ECL, Thermo, USA). The expression levels of target genes were normalized to the levels of GAPDH or β-actin within each sample. Each experiment was conducted independently at least three times.

### 2.7. Dual luciferase reporter assay

STK3/4 (STK3 in rat and STK4 in human) were predicted as the target gene of miR-301a-3p using Targetscan 3.1 and miRanda 3.3a. To validate the interaction between miR-301a-3p and STK3/4, a dual luciferase reporter assay was performed. Firstly, a fragment within the 3′UTR of STK3/4, containing the predicted miR-301a-3p target site, was amplified from OMECs cDNA using PCR primers. Subsequently, the amplified product was inserted into the SacI and XhoI restriction sites of a psi-check2 vector, generating a wild-type psi-check2 dual luciferase reporter vector. Additionally, complementary mutations were introduced into the miR-301a-3p target binding sequences within the 3′UTR of STK3/4, resulting in the production of a mutant psi-check2 dual luciferase reporter vector. Validation of the constructed vectors was performed through electrophoresis on 1.5% agarose gels and Sanger sequencing.

Subsequently, 0.20 μg of plasmid was extracted from both the wild-type and mutant psi-check2 dual luciferase reporter vectors. These plasmids were co-transfected with 20 pmol of either miR-301a-3p mimic or miR-301a-3p mimic NC into HEK293T cells using INVI DNA & RNA Transfection Reagent™ (Invigentech, CA, USA). Following transfection for 48 hours, the Renilla and firefly luciferase activities of STK3/4 were measured using the Dual-Luciferase Reporter Kit (Promega, WI, USA) and analyzed with a Varioskan LUX Microplate Reader (Thermo Lifetech, MA, USA).

### 2.8. Chromatin immunoprecipitation assay

PDAC cells (2 × 106) were subjected to chromatin immunoprecipitation (ChIP) assay using a ChIP assay kit (Millipore) following the manufacturer’s instructions. The precipitated DNA samples obtained from the ChIP assay were subsequently subjected to PCR amplification targeting specific regions of the STK3/4 promoter. The resulting PCR products were then separated by electrophoresis on a 2% agarose gel and visualized using ethidium bromide staining.

### 2.9. Cell cycle assay

Cells were collected 48 hours post-infection, washed, and suspended in 0.1 M PBS. Subsequently, the cells were fixed in 1 ml pre-cooled 70% alcohol overnight at 4°C, with a cell concentration of 1.0 × 106 cells/ml. After fixation, the cells were treated with a staining solution containing 50 μg/ml propidium iodide (PI) and 20 μg/ml RNase A for 30 minutes at 4°C in the absence of light. The cell cycle distribution was then assessed using flow cytometry.

### 2.10. Transwell cell invasion assay

A total of 2 × 104 cells were seeded in 200 μL of serum-free DMEM into the upper chamber of a 24-well transwell system (Matrigel Invasion Chamber, BD 354,480) for invasion assays. In the lower chamber, 600 μL of DMEM supplemented with 10% FBS was added. After incubation for 60 or 72 hours, the inserts were removed, and the cells on the upper surface were gently washed with PBS and removed using cotton swabs. Subsequently, the cells were fixed in 100% ethanol for 20 minutes and stained with crystal violet. The stained inserts were then cut and mounted on microscope slides. Six fields were randomly selected from each insert for cell counting. Images of the invading cells were captured using an inverted microscope (Olympus). Each experiment was repeated independently three times.

### 2.11. Cell counting Kit-8 assay

Cell viability was assessed using the Cell Counting Kit-8 Assay Kit (Dojindo, Japan). Briefly, cells were seeded into 96-well plates at a density of 2 × 104 cells per well and allowed to adhere overnight. Subsequently, cells were treated with various conditions, including 1% FBS-DMEM, 100 ng/mL Shh, 5 ng/mL IL-1β, 10 ng/mL TNF-α, or miR-301a inhibitor alone or in combination. At 0, 12, 24, 36, and 48 hours post-stimulation, cells were incubated with 2-(2-methoxy-4-nitrophenyl)-3-(4-nitrophenyl)-5-(2,4-disulfophenyl)-2 h-tetrazolium monosodium salt (WST-8) in the dark as per the manufacturer’s instructions. The absorbance values were measured at 450 nm using an enzyme-linked immunosorbent assay (ELISA) reader. Each experiment was performed in triplicate to ensure reproducibility.

### 2.12. Apoptosis assay

Apoptosis was assessed using the Annexin V/FITC staining kit (eBioscience, USA). At 48 hours post-stimulation, cells were harvested and washed twice with PBS. Subsequently, the cells were centrifuged, resuspended in 100 μL of binding buffer, and stained with Annexin V (5 μL) for 15 minutes at room temperature in the dark. Following Annexin V staining, the samples were stained with propidium iodide (10 μL) for 5 minutes in the dark before being subjected to flow cytometry (Miltenyi Biotec, Germany). The acquired data were analyzed using FlowJo software (Tree Star Inc., USA).

### 2.13. Statistical analysis

Statistical analyses were performed using the Statistical Package for the Social Sciences (SPSS, version 18.0; USA). Student’s t-test was employed to compare groups and determine statistical significance. Pearson’s correlation coefficient was calculated to assess correlations. All data are presented as mean ± standard deviation. A value of *p* < .05 was considered statistically significant.

## 3. Results

### 3.1. Activation of hedgehog pathway regulates the Hippo/YAP pathway

In our previous studies, we found cytokines TNF-α and IL-1β can activate the Hh pathway.^18,19^ To explore the influence of the Hedgehog pathway on the Hippo/YAP pathway and unravel the underlying mechanisms, Panc-1 cells were treated separately with the YAP pathway inhibitor verteporfin, SHH protein (20, 100, and 500 ng/mL), TNF-α (0.5, 5, and 50 ng/mL), and IL-1β (0.5, 5, and 50 ng/mL). Following these treatments, the expression levels and phosphorylation status of key molecules within the YAP pathway were evaluated.

**Figure 1.**
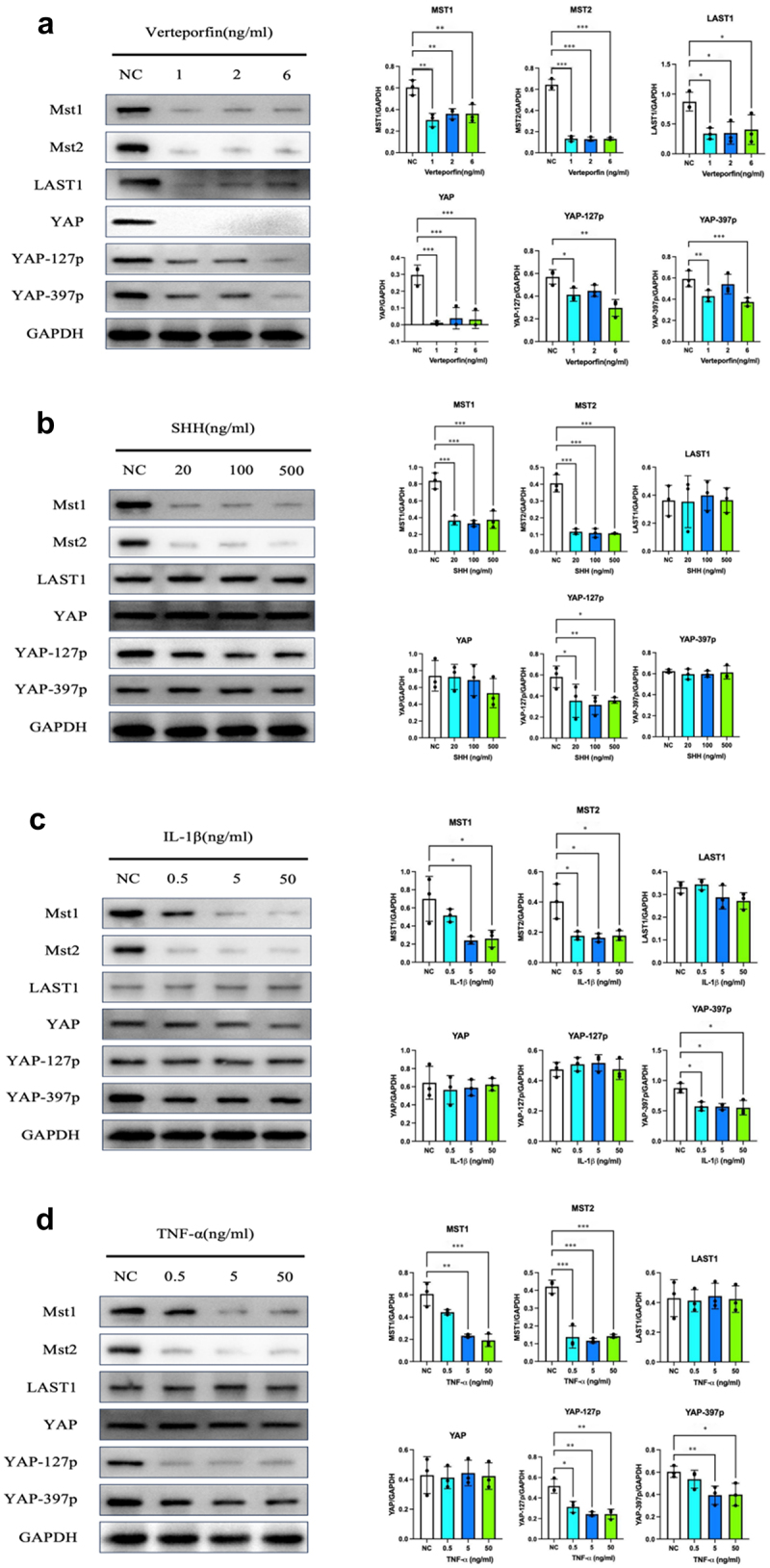
Regulation of the Hippo/yap pathway by hedgehog activation in panc-1 cells. Protein expression and phosphorylation levels of Hippo/yap pathway members in panc-1 cells after 48-hour treatment with the YAP pathway inhibitor verteporfin (a), SHH (b), tnf-α (c), and IL-1β (d). Data are presented as mean ± SD, *n*=3; *p < .05, **p < .01,***p < .001 compared to controls. Statistical analyses used one-way ANOVA with post hoc tests.

Treatment with verteporfin led to a significant decrease in the expression of Mst1 and Mst2, as well as in the downstream molecules LATS1 and total YAP protein, indicating a nonspecific inhibition of the YAP pathway (Figure 1a). In contrast, SHH treatment resulted in reduced expression of YAP-127p, Mst1, and Mst2, while the levels of total YAP, LATS1, and YAP-397p remained unchanged (Figure 1b). Similarly, IL-1β treatment decreased the expression of YAP-397p, Mst1, and Mst2, without affecting total YAP, LATS1, or YAP-127p levels (Figure 1c). TNF-α treatment also led to a reduction in YAP-127p, YAP-397p, Mst1, and Mst2 expression, with no significant changes observed in total YAP and LATS1 levels (Figure 1d).

The localization and activity of YAP are regulated by phosphorylation at specific inhibitory sites, where phosphorylation at Ser127 and Ser397 suppresses YAP activation and thereby inhibits the Hippo/YAP pathway.^20^ Phosphorylation at Ser127 retains YAP in the cytoplasm, preventing its nuclear entry and transcriptional activity, while phosphorylation at Ser397 targets YAP for degradation, reducing its overall cellular levels.^21^ In this study, we found that activation of the Hedgehog pathway by SHH, TNF-α, and IL-1β significantly reduced phosphorylation at these sites, promoting YAP nuclear translocation. This shift enhances YAP pathway activity by downregulating the upstream regulators MST1 and MST2, ultimately impacting processes such as cell proliferation, differentiation, and apoptosis. These findings suggest that Hedgehog pathway activation promotes YAP nuclear localization and pathway activation by decreasing inhibitory phosphorylation and suppressing MST1 and MST2 expression.

### 3.2. miR-301a is the most upregulated miRNA upon hedgehog activation, and its precursor directly binds to Gli1

**Figure 2.**
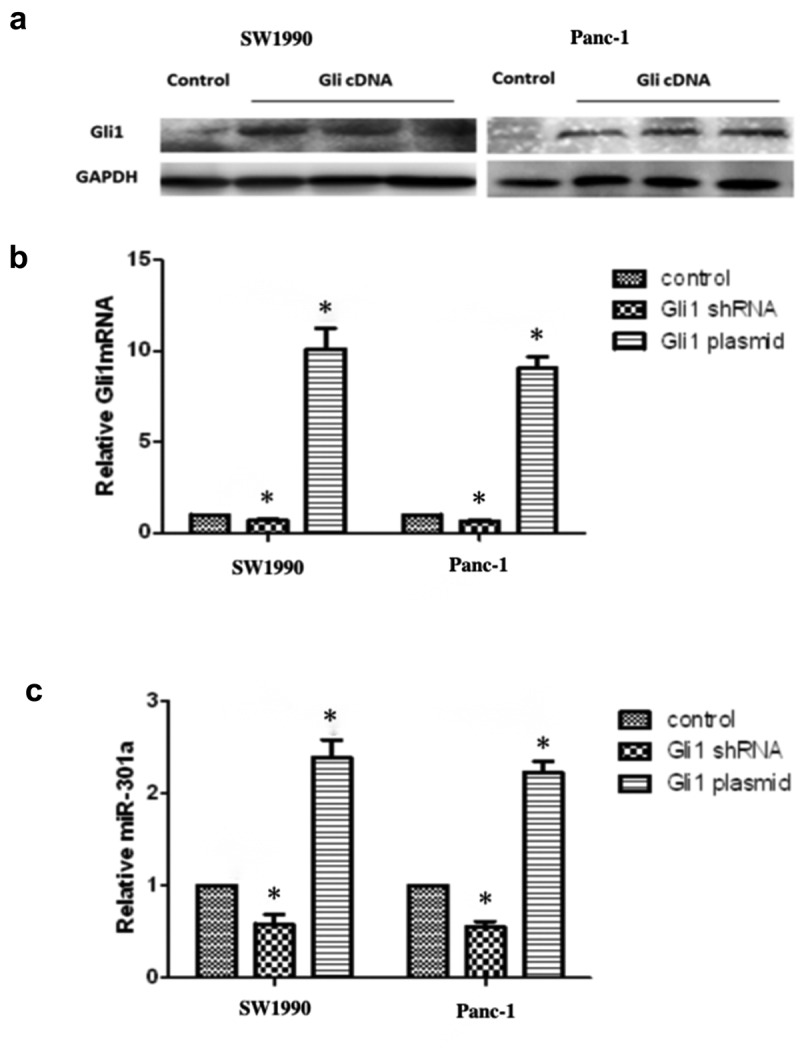
Activation of hedgehog signaling via Gli1 overexpression and its effects on mi301a expression in SW1990 and panc-1 cells. (a) Western blot analysis showing Gli1 protein expression in SW1990 and panc-1 cells following transfection with Gli1 cDNA to activate hedgehog (HH) signaling. (b) Quantitative RT-PCR analysis of Gli1 mRNA levels after transfection with either Gli1 shRNA (knockdown) or Gli1 plasmid (overexpression) in SW1990 and panc-1 cells, demonstrating effective modulation of Gli1 expression. (c) Expression of miR-301a in response to Gli1 knockdown or overexpression, indicating downstream effects of HH pathway activation on miRNA regulation. Data are presented as mean ± SD, *n*=3; with *indicating a statistically significant difference compared to the control group (*p* < .05). Statistical analyses were performed using one-way ANOVA followed by post hoc tests for multiple comparisons, as indicated by the p-values on each graph.

To investigate the influence of Gli1 expression on miR-301a regulation, we transfected Gli1 cDNA expression vectors and Gli1 shRNA knockout vectors into SW1990 and Panc-1 cells individually. Consistent alterations in Gli1 protein and mRNA expression levels were observed in both cell lines (Figure 2a-b). Notably, alongside these changes, we observed significant shifts in miR-301a expression, notably increasing upon Gli1 overexpression and decreasing upon Gli1 knockout, further indicating miR-301a as a downstream target of Gli1 (Figure 2c).

**Figure 3.**
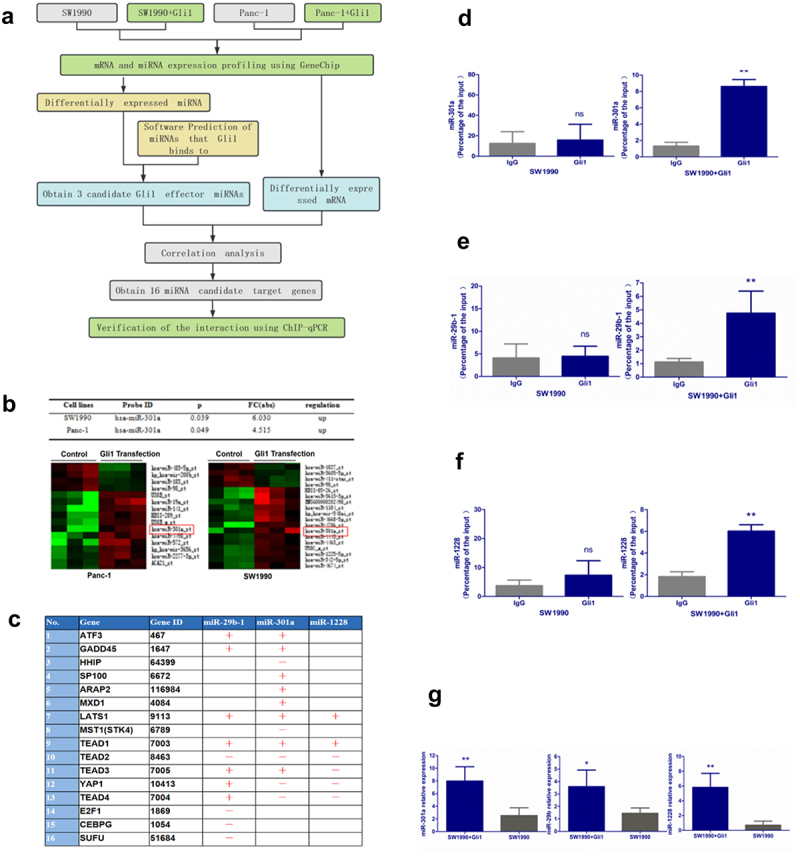
Differential expression of miRNA following transfection with Gli1 cDNA. (a) Experimental flow chart of gene chip screening for differentially expressed miRNA after transgenic Gli1cDNA in pancreatic cancer cell lines. (b) miR-301a is highly expressed in SW1990 and panc-1 cells after transfection with Gli1cDNA. (c) Summary of the correlation between 3 differentially expressed miRNAs (miR-301a, miR-1228 and miR-29b) and 16 differentially expressed genes after Gli1 cDNA transfection.(d-f) chip-qPCR verified the presence of Gli1 nuclear factor binding sites in the precursor promoters of miR-301a, miR-1228 and miR-29b. (g) Gli1 overexpression can significantly promote the expression of its target genes, miR-301a, miR-1228 and miR-29b.Data are presented as mean ± SD, *n* = 3; *indicates a statistically significant difference compared to the control group (*p* < .05), and **indicates a highly significant difference (*p* < .01). Statistical analyses were performed using one-way ANOVA followed by post hoc tests for multiple comparisons.

Subsequently, we independently transfected SW1990 and Panc-1 cells with Gli1 cDNA for mRNA and miRNA microarray analysis (Figure 3a). The results unveiled significant differential expression patterns, with numerous miRNAs and mRNAs showing changes post-Gli1 transfection. Remarkably, miR-301a exhibited high expression in both SW1990 and Panc-1 cells transfected with Gli1 cDNA (Figure 3b).

**Figure 4.**
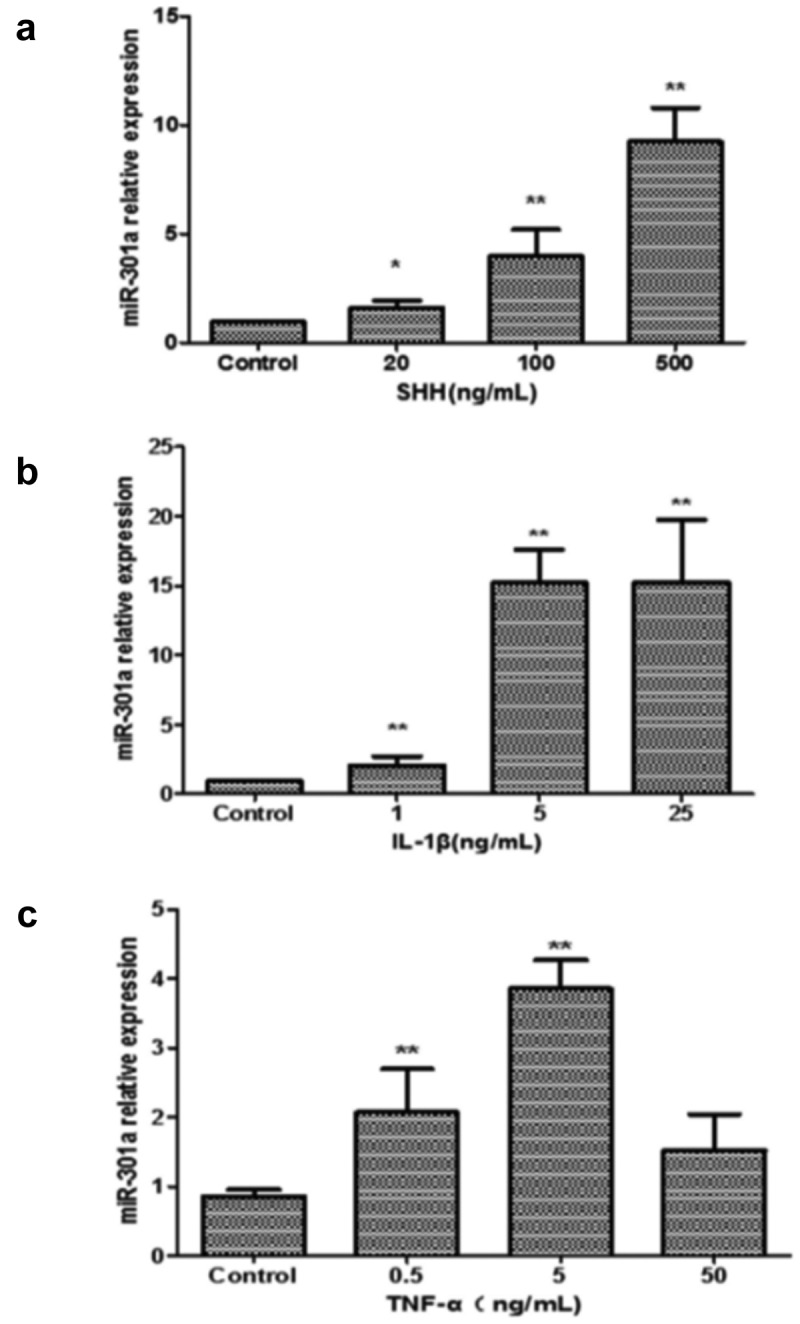
Upregulation of miR-301a expression by hedgehog activator shh and inflammatory factors IL-1β and tnf-α. (a) Relative expression of miR-301a in response to sonic hedgehog (shh) treatment at varying concentrations (20, 100, and 500 ng/mL), showing a dose-dependent increase compared to the control group. (b) miR-301a expression following treatment with IL-1β at concentrations of 1, 5, and 25 ng/mL, demonstrating significant upregulation in response to increased IL-1β. (c) miR-301a expression after tnf-α treatment at 0.5, 5, and 50 ng/mL, with a peak increase at 5 ng/mL. Data are shown as mean ± SD, with * indicating *p* < .05 and **indicating *p* < .01 compared to the control group. Statistical analyses were performed using one-way ANOVA followed by post hoc tests for multiple comparisons.

Moving forward, Panc-1 cells were exposed to varying concentrations of SHH, IL-1β, and TNF-α proteins, and changes in miR-301a expression were monitored within 24 hours (Figure 4). Notably, SHH stimulation led to a significant increase in miR-301a expression levels, while IL-1β also triggered a notable elevation in miR-301a expression. In contrast, TNF-α induced a significant rise in miR-301a expression at a lower concentration (5 ng/mL), but this effect diminished at a higher concentration (50 ng/mL).

### 3.3. miRNA-301a significantly suppresses the expression of MST1 (STK4), SHH and directly targets STK4

**Figure 5.**
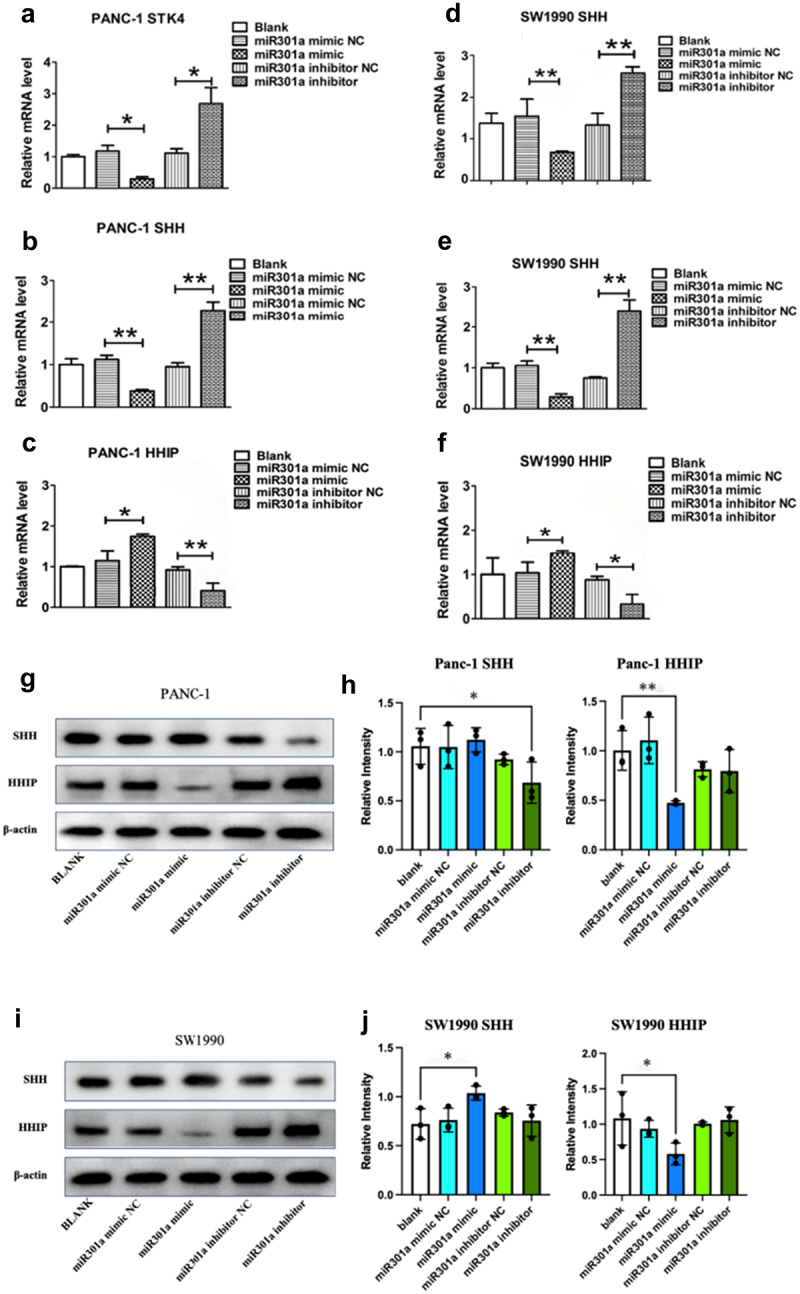
Regulatory effects of miR-301a on MST1 (STK4), SHH, and HHIP mRNA and protein expression levels in pancreatic cancer cell lines. (a-f) quantitative PCR analysis of mRNA levels in panc-1 and SW1990 cells. miR-301a mimics significantly downregulate the mRNA expression of MST1 and SHH while upregulating HHIP expression. miR-301a inhibitors exhibit the opposite effects, increasing MST1 and SHH mRNA levels while decreasing HHIP expression. (g-j) Western blot analysis confirms that miR-301a mimics reduce SHH protein levels and increase HHIP protein levels in both panc-1 and SW1990 cells, while miR-301a inhibitors lead to the opposite pattern of regulation. Data are presented as mean ± SD, *n* = 3, with statistical significance indicated (*p < .05, **p < .01 compared to respective controls). Statistical analyses were performed using one-way ANOVA followed by post hoc tests for multiple comparisons.

To investigate the regulatory role of miR-301a in pancreatic cancer cell lines SW1990 and Panc-1, synthetic miR-301a mimics and inhibitors were transfected into these cells, and the mRNA and protein expression levels of MST1 (STK4), SHH, and HHIP were analyzed. In Panc-1 cells (Figure 5a-c), the miR-301a mimic significantly downregulated the mRNA levels of MST1 (STK4) and SHH while significantly upregulating the expression of HHIP. Conversely, the miR-301a inhibitor increased MST1 (STK4) and SHH mRNA levels and reduced HHIP expression. A similar trend was observed in SW1990 cells (Figure 5d-f), where the miR-301a mimic reduced SHH mRNA levels and increased HHIP expression, while the inhibitor exhibited opposite effects. At the protein level, Western blot analysis demonstrated that in Panc-1 cells, miR-301a significantly decreased SHH protein expression (Figure 5g-h) and upregulated HHIP expression. In SW1990 cells (Figure 5i-j), the miR-301a mimic similarly led to a reduction in SHH protein expression and an increase in HHIP expression, while the miR-301a inhibitor produced opposite regulatory effects in both cell lines. These findings indicate that miR-301a modulates the expression of key components of the Hedgehog signaling pathway, potentially leading to the activation of this pathway in pancreatic cancer cells.

**Figure 6.**
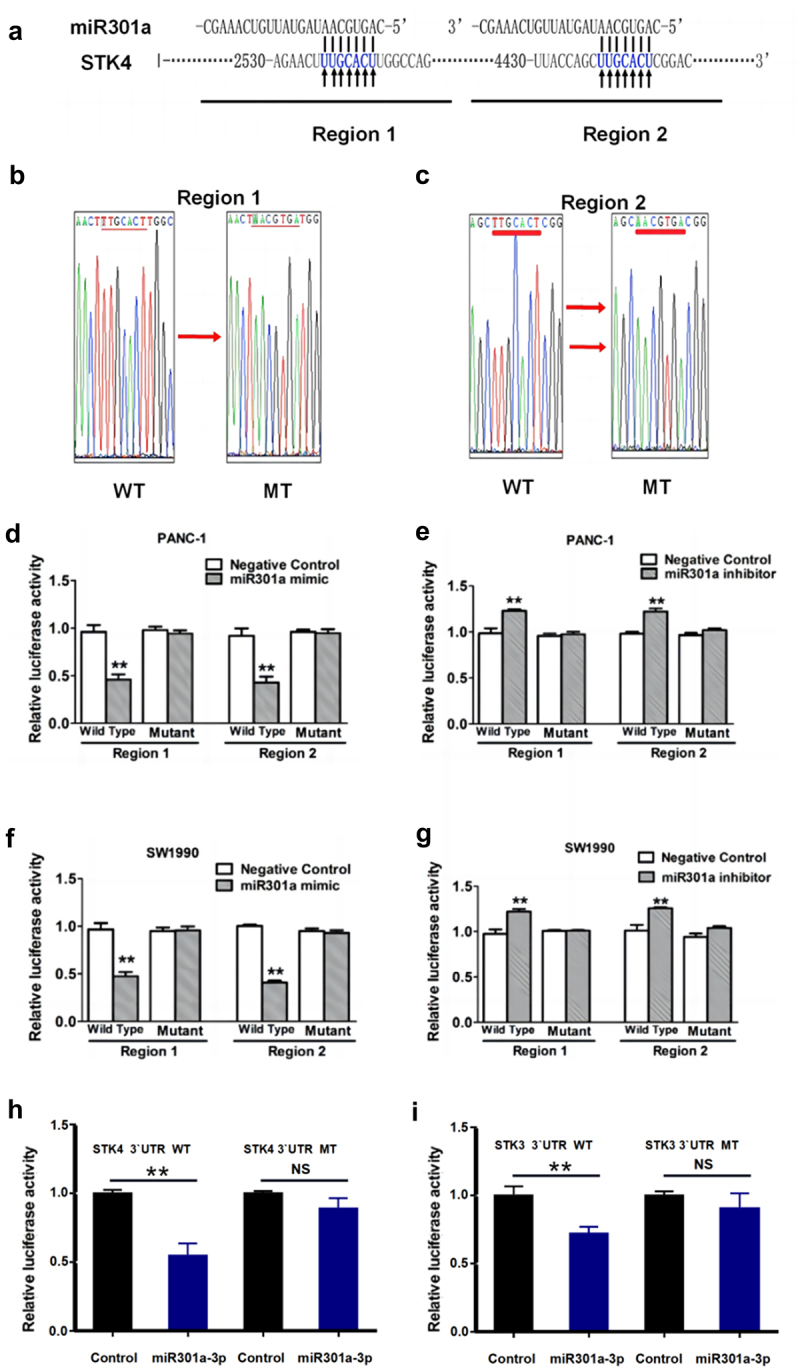
miR-301a directly targets and regulates MST1. (a-c) the 3’-UTR of the STK4 gene contains two regions with binding sites for miR-301a. Wild-type (WT) and mutant (MT) reporter constructs for these regions (region 1 and region 2) were created. (d-g) the reporter gene activity was measured in PANC-1 and SW1990 cells co-transfected with miR-301a binding sequences and mutant constructs using miR-301a mimic or inhibitor. (h-i) the regulatory effect of miR-301a on human STK4 and rat STK3 3’-UTR was further assessed through a dual-luciferase reporter assay. Data are presented as mean ± SD, *n* = 3, with **indicating a statistically significant difference (*p* < .01) compared to controls. Statistical analyses were performed using one-way ANOVA with post hoc tests for multiple comparisons.

Moreover, we constructed wild-type (WT) and mutant reporter gene vectors containing miR-301a binding sequences 1 (region 1) and 2 (region 2) of the Mst1 (STK4) gene’s 3‘UTR (Figure 6a-c). These vectors were transfected into pancreatic cancer cell lines Panc-1 and SW990. Co-transfection with miR-301a mimics significantly suppressed reporter gene expression, while co-transfection with miR-301a inhibitors led to upregulation of reporter gene expression (Figure 6d-g). Similar results were observed in both cell lines.

Further analysis using TargetScan revealed miR-301a’s predominant regulation of STK4 in human cells and STK3 in mice. Plasmids carrying human STK4 3‘UTR sequence and its mutant, as well as mouse STK3 3‘UTR sequence and its mutant, were constructed for subsequent experiments. Co-transfection with miR-301a into 293T cells showed direct binding and inhibition of the expression of human STK4 and mouse STK3 3‘UTR sequences (Figures 6h-i). This preparation sets the stage for subsequent animal experiments.

### 3.4. Inhibition of miR-301a suppresses pancreatic cancer cell proliferation and promotes apoptosis

**Figure 7.**
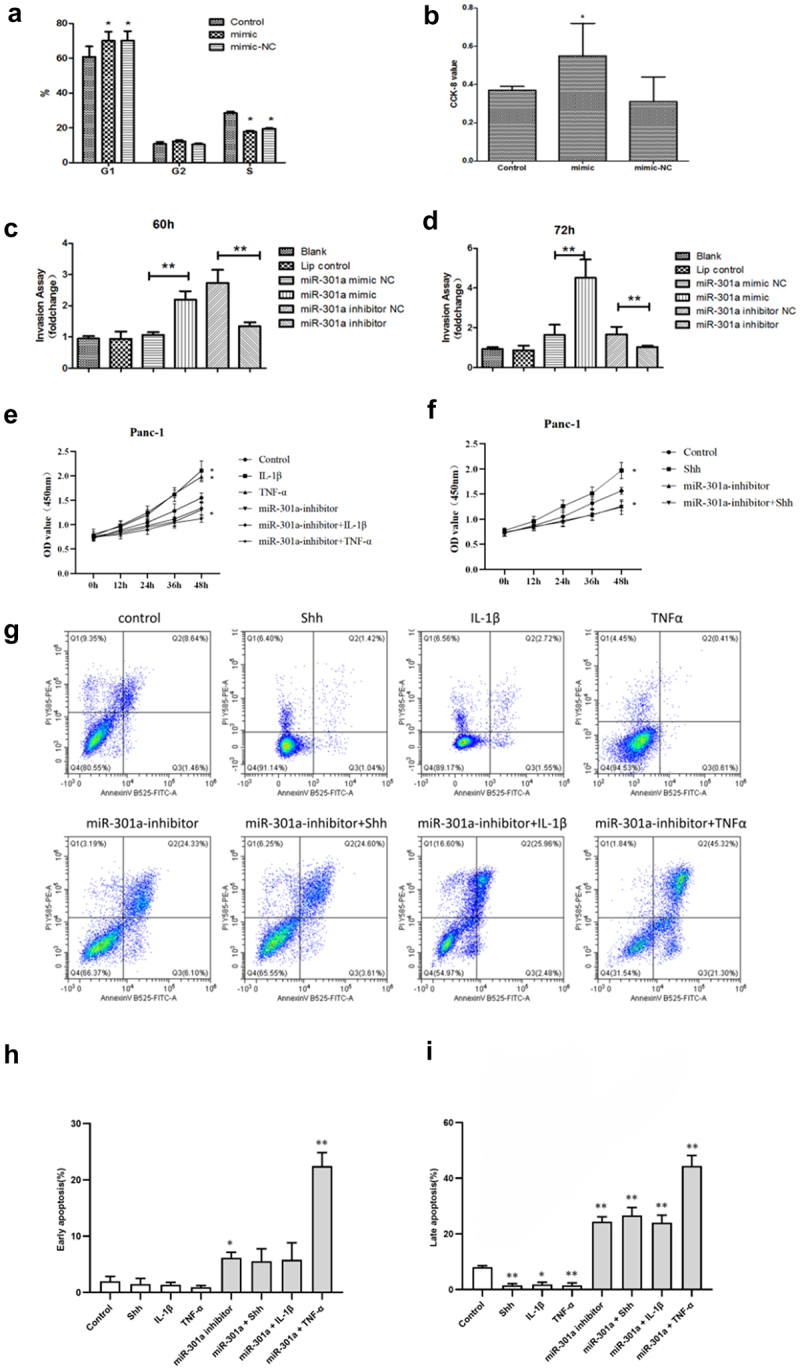
Impact of miR-301a modulation on cell cycle, proliferation, invasion, and apoptosis in PANC-1 cells. (a) miR-301a mimic increased the G1 phase and decreased the S phase in cell cycle distribution. (b) CCK-8 assay showed enhanced proliferation with miR-301a mimic. (c-d) miR-301a mimic promoted cell invasion, while miR-301a inhibitor reduced it. (e-f) proliferation increased over time under various treatments, especially with inflammatory stimuli. (g-i) miR-301a inhibitor, especially combined with SHH, IL-1β, or tnf-α, significantly elevated early and late apoptosis rates. Data are presented as mean ± SD; *p < .05, **p < .01 compared to controls. Statistical analyses used one-way ANOVA with post hoc tests.

In Panc-1 cells, preliminary experimental results from increased endogenous miR-301a expression indicate a significant reduction in the G1 and G2 phases (Figure 7a), accompanied by enhanced cell proliferation (Figure 7b). Furthermore, invasion experiments revealed that treatment with miR-301a mimics and inhibitors for 60 and 72 hours significantly promoted cell invasion compared to the control group (Figure 7c,d).

Subsequently, Panc-1 cells were exposed to 100 ng/ml Shh, 5 ng/ml IL-1β, and 10 ng/ml TNF-α, along with a miR-301a inhibitor, to assess changes in cell proliferation and apoptosis. The results demonstrated that treatment with Shh, IL-1β, and TNF-α alone increased cell proliferation compared to the blank control group, while reducing the number of apoptotic cells. However, after treatment with the combination of miR-301a inhibitor, the enhanced proliferation capacity weakened, even falling below that of the control group, and exhibited a synergistic effect with TNF-α (Figure 7e,f). This was accompanied by a significant increase in the number of apoptotic cells (Figure 7g,h,i). These findings suggest that the miR-301a inhibitor effectively suppresses cell proliferation and promotes mainly late apoptosis, indicating its potential to counteract the promoting effects of SHH, IL-1β, and TNF-α on the malignant characteristics of cells.

## 4. Discussion

Despite the major advances in uncovering the molecular mechanisms underlying pancreatic ductal adenocarcinoma (PDAC), this disease remains one of the most lethal human cancers, with limited effective therapeutic options.^22^ The Hedgehog (Hh) and HIPPO/YAP signaling pathways play pivotal roles in PDAC initiation and progression, each governing critical aspects of cell proliferation, survival, and tissue growth.^23,24^ Dysregulation in these pathways promotes aberrant cellular behavior that contributes significantly to tumor aggressiveness. Our study has identified miR-301a as a central regulatory node between the Hh and HIPPO/YAP pathways, directly targeting key molecules within each pathway, including Gli1 in Hh signaling and STK4 (MST1) in the HIPPO/YAP pathway. This dual targeting allows miR-301a to exert considerable influence over pathway crosstalk, ultimately affecting PDAC pathogenesis.

In the context of Hh signaling, miR-301a modulates pathway activity through its regulation of Gli1, which is essential for downstream signal transduction and gene activation associated with cell proliferation and tumor growth. By binding to Gli1, miR-301a amplifies Hh pathway signaling, as evidenced by increased expression of target genes such as SHH and HHIP. In addition to its regulation of Hh signaling, miR-301a simultaneously influences the HIPPO/YAP pathway by targeting STK4, a kinase critical to YAP phosphorylation and nuclear-cytoplasmic shuttling. Reduced STK4 expression leads to decreased phosphorylation of YAP, allowing YAP to translocate to the nucleus where it interacts with transcription factors to activate genes that promote cell proliferation and survival. Our study highlights that miR-301a-mediated suppression of STK4 reduces YAP phosphorylation, thereby enhancing YAP activity within the nucleus, which promotes oncogenic processes in PDAC cells.

Our experiments further demonstrate that inflammatory cytokines, such as TNF-α and IL-1β, alongside Sonic Hedgehog (SHH) ligand, can stimulate miR-301a expression. This upregulation suggests a feedback loop where pro-inflammatory signals in the tumor microenvironment potentiate miR-301a activity, thereby reinforcing both Hh and HIPPO/YAP pathway dysregulation. This mechanism is particularly noteworthy, as inflammatory cytokines like TNF-α are commonly elevated in PDAC and are associated with disease progression.^25^ The ability of miR-301a to respond to such stimuli positions it as a key modulator linking inflammation and cancer signaling, adding another layer to its role in PDAC pathogenesis.

To further elucidate the effects of miR-301a on tumor cell behavior, we performed functional assays examining cell proliferation, apoptosis, and invasion. The results confirm that miR-301a promotes PDAC cell proliferation and inhibits apoptosis. In particular, miR-301a inhibition was shown to enhance apoptosis significantly, especially in the presence of TNF-α, suggesting a synergistic effect between miR-301a suppression and inflammatory signaling. Additionally, in vitro invasion assays revealed that miR-301a increased the invasive potential of PDAC cells, highlighting its role in facilitating metastatic progression. Our conclusion that miR-301a promotes pancreatic cancer progression is also supported by previous reports, which have demonstrated this role through different pathways.^26,27^ Targeting miR-301a directly or modulating its interaction with Gli1 and STK4 offers a promising strategy to disrupt the pathological signaling driving PDAC. Given the ability of miRNAs to target multiple pathways, inhibiting miR-301a could yield broader therapeutic benefits compared to targeting single proteins within these pathways.

Our study sheds light on the intricate interplay among GLI1, MST1, and miR-301a, which connects the Hedgehog (Hh) and HIPPO/YAP signaling pathways in pancreatic ductal adenocarcinoma (PDAC). We demonstrated that GLI1, a key effector of the Hh pathway, transcriptionally upregulates miR-301a by binding to its promoter region. In turn, miR-301a directly targets STK4 (MST1), a core component of the HIPPO pathway, leading to the suppression of MST1 expression and subsequent activation of YAP. This regulatory axis is further amplified under cytokine stimulation, highlighting the role of microenvironmental cues in modulating these interactions. While this study establishes a robust framework for understanding these interactions, additional studies are needed to further delineate their temporal dynamics, in vivo relevance, and potential implications for therapy resistance in PDAC. Future research could explore how this axis responds to diverse microenvironmental stimuli or contributes to the aggressiveness of PDAC.

In summary, this study elucidates the intricate regulatory mechanisms of miR-301a in modulating Hh and HIPPO/YAP pathways in PDAC. Our data highlight miR-301a as a critical regulator facilitating pathway crosstalk, directly impacting PDAC cell proliferation, survival, and metastasis. The dual role of miR-301a in targeting Gli1 and STK4 positions it as a unique molecular switch that amplifies oncogenic signaling cascades in PDAC. These findings open new avenues for therapeutic intervention, and future studies focusing on miR-301a inhibitors or delivery systems targeting PDAC cells could provide promising strategies for combating this aggressive cancer.

## Highlights

- miR-301a is the most upregulated miRNA when Hedgehog is activated and its precursor directly binds to Gli1.
- miR-301a significantly suppresses the expression of MST1 (STK4), SHH, HHIP and directly targets STK4.
- Inhibition of miR-301a suppresses pancreatic cancer cell proliferation and promotes apoptosis.

## Data Availability

Microarray analysis for mRNA and miRNA data is available at the Gene Expression Omnibus (GEO; GSE281315, GSE281316). Any additional information regarding data reported in this article is available from the corresponding author upon request. There are no restrictions on data availability, and no custom code was used to analyze data in this study.

## Abbreviations

PDAC: pancreatic ductal adenocarcinoma
YAP: yes-associated protein
Gli1: glioma-associated oncogene homolog 1
SHH: sonic hedgehog
MST1/2: mammalian Ste20-like kinases ½
LAST1/2: large tumor suppressor kinases ½
HHIP: hedgehog-interacting protein
